# Italian Age of Acquisition Norms for a Large Set of Words (ItAoA)

**DOI:** 10.3389/fpsyg.2019.00278

**Published:** 2019-02-13

**Authors:** Maria Montefinese, David Vinson, Gabriella Vigliocco, Ettore Ambrosini

**Affiliations:** ^1^Department of Experimental Psychology, University College London, London, United Kingdom; ^2^Department of General Psychology, University of Padua, Padua, Italy; ^3^Department of Neuroscience, University of Padua, Padua, Italy

**Keywords:** age of acquisition, word, lexicon, Italian language, cross-linguistic comparison, subjective rating

## Abstract

Age of acquisition (AoA) is an important psycholinguistic variable that affects the performance of healthy individuals and patients in a large variety of cognitive tasks. For this reason, it becomes more and more compelling to collect new AoA norms for a large set of stimuli in order to allow better control and manipulation of AoA in future research. An important motivation of the present study is to extend previous Italian norms by collecting AoA ratings for a much larger range of Italian words for which concreteness and semantic-affective norms are now available thus ensuring greater coverage of words varying along these dimensions. In the present study, we collected AoA ratings for 1,957 Italian content words (adjectives, nouns, and verbs), by asking healthy adult participants to estimate the age at which they thought they had learned the word in a Web survey procedure. First, we found high split-half correlation within our sample, suggesting strong internal reliability. Second, our data indicate that the ratings collected in this study are as valid and reliable as those collected in previous studies for Italian across different age populations (adult and children) and other languages. Finally, we analyzed the relation between AoA ratings and other lexical-semantic variables (e.g., word frequency, imageability, valence, arousal) and showed that these correlations were generally consistent with the correlations reported in other normative studies for Italian and other languages. Therefore, our new AoA norms are a valuable source of information for future research in the Italian language. The full database is available at the Open Science Framework (osf.io/3trg2).

## Introduction

Age of acquisition (AoA) represents the age at which a word is learned. This measure has been shown to affect performance in a large variety of cognitive tasks (see reviews by Juhasz, 2005; Johnston and Barry, 2006; Brysbaert and Ellis, 2016), with faster reaction times for words learned early in life compared with those learned later. In particular, AoA affects lexical tasks, such as picture naming (Navarrete et al., 2015), written word naming (Brysbaert et al., 2000a), lexical decision (Cortese and Khanna, 2007) and semantic tasks, such as word association and semantic categorization (Brysbaert et al., 2000b). Moreover, an advantage for early-learned words is also seen in response accuracy in verbal tasks in several neuropsychological disorders, such as, Alzheimer’s disease (Silveri et al., 2002; Sartori et al., 2005), aphasia (Cuetos et al., 2002), dysgraphia (Weekes et al., 2003), and dyslexia (Barry and Gerhand, 2003). Importantly, such AoA effects have been observed in a wide variety of languages including English (Lambon Ralph and Ehsan, 2006), Italian (Bates et al., 2001; Menenti and Burani, 2007; Navarrete et al., 2013), Chinese (Chen et al., 2007), Turkish (Raman et al., 2014), French (Bonin et al., 2004), and Dutch (Brysbaert et al., 2000a; Menenti and Burani, 2007).

Connectionist models provide different accounts by which AoA may affect processing. The network plasticity hypothesis suggests that learning of new words is not constant and is accompanied by a gradual decline over time in the plasticity of the network responsible for learning patterns and associations, resulting in less efficient learning for later-acquired words (Ellis and Lambon Ralph, 2000). This general account of AoA effect as not specific of a particular domain (e.g., orthography, phonology, semantics) is compatible with aspects of several theoretical frameworks. For example, the phonological completeness view posits that early-acquired words have more complete phonological representations than later-acquired words and form a foundation for the less complete words learned later in life (Brown and Watson, 1987). Similarly, the semantic locus view suggests that early-acquired words help to build the semantic network for the acquisition of later-learned words (Steyvers and Tenenbaum, 2005). Thus, words with lower AoA have more connections and are used more often compared to later-acquired words, making their retrieval easier (see the cumulative frequency hypothesis by Zevin and Seidenberg, 2004).

Because AoA is correlated with a number of other lexical-semantic variables, the extent to which AoA affects processing independently of other variables has been contested. Indeed, earlier-acquired words tend to be associated with higher frequency and familiarity values, both of which also facilitate processing (Morrison and Ellis, 2000). Similarly, controversy has arisen about whether effects of AoA may be explained in terms of imageability, as the two are moderately correlated (Cortese and Khanna, 2007). Finally, AoA is also correlated to affective measures: words that are learned early in life are rated as more positive and less dominant (Moors et al., 2013).

To test whether AoA effects can be observed independently of effects of other lexical-semantic variables, it is thus necessary to collect AoA estimates and other lexical-semantic measures for word stimuli and make them readily available. There are two main approaches to derive AoA data. First, objective AoA measures can be obtained by analysis of children’s production (Chalard et al., 2003; Álvarez and Cuetos, 2007; Lotto et al., 2010; Grigoriev and Oshhepkov, 2013). Within this approach, children (classified by age) are asked to name the picture of common objects and activities. The AoA of a given word is computed as the mean age of the group of children in which at least 75% of them can name the picture correctly. Alternatively, subjective AoA can be obtained by using adult estimates (Barca et al., 2002; Ferrand et al., 2008; Moors et al., 2013). Here, adult participants are asked to provide ratings of AoA on either a Likert scale (Schock et al., 2012; Alonso et al., 2015; Borelli et al., 2018) or directly in years, by indicating the number corresponding to the age they thought they had learned a given word (Stadthagen-Gonzalez and Davis, 2006; Ferrand et al., 2008; Moors et al., 2013). Compared to the use of a Likert scale, this latter method is easier for participants to use and it does not restrict the response range artificially, instead providing more precise information about the words’ AoA (Ghyselinck et al., 2000). It has been shown that the AoA estimates obtained from the two different methods are highly correlated (Morrison et al., 1997; Ghyselinck et al., 2000; Pind et al., 2000; Lotto et al., 2010; see also Brysbaert, 2017; Brysbaert and Biemiller, 2017) and this correlation still remains significant when other variables, such as familiarity, frequency, and phonological length, are controlled for (Bonin et al., 2004).

Subjective AoA ratings for a large set of content words (adjectives, nouns, and verbs) varying substantially in concreteness are now available for English (Kuperman et al., 2012), Spanish (Alonso et al., 2015), Portuguese (Cameirão and Vicente, 2010), Dutch (Moors et al., 2013), and French (Ferrand et al., 2008). For Italian, however, the vast majority of both objective and subjective AoA ratings have been gathered only for nouns (Dell’Acqua et al., 2000; Barca et al., 2002; Barbarotto et al., 2005; Della Rosa et al., 2010) and concrete words (Dell’Acqua et al., 2000; Barca et al., 2002; Barbarotto et al., 2005; Lotto et al., 2010). Only two sets of Italian norms with objective AoA (Rinaldi et al., 2004) and subjective AoA (Borelli et al., 2018) include abstract and concrete words and different word classes (adjective, noun, and verb), but they are limited to a relatively small number of word stimuli (519 and 512 words, respectively). Unfortunately, the lack of overlap between AoA (Dell’Acqua et al., 2000; Barca et al., 2002; Barbarotto et al., 2005; Della Rosa et al., 2010; Borelli et al., 2018) and semantic-affective norms (Zannino et al., 2006; Kremer and Baroni, 2011; Montefinese et al., 2013b, 2014; Fairfield et al., 2017) for Italian words has prevented the direct comparison of different lexical-semantic dimensions to establish the extent to which they overlap or complement each other in word processing.

In the present paper, we provide subjective AoA ratings for a large set of Italian words (1,957 content words: adjective, nouns, and verbs), with a wide range of concreteness. Words were chosen to overlap as much as possible with other Italian norms (Zannino et al., 2006; Kremer and Baroni, 2011; Fairfield et al., 2017; Montefinese et al., 2013b, 2014). In addition, to allow assessment of the reliability of AoA measure obtained in the current norms, we selected stimuli from previous Italian subjective and objective AoA norms (Dell’Acqua et al., 2000; Barca et al., 2002; Rinaldi et al., 2004). Finally, we also provide other lexical measures related to the AoA of words and we explore the relation between AoA and other linguistic and semantic variables known to influence the processing of word meaning (Montefinese and Vinson, 2015; Montefinese et al., 2015).

## Materials and Methods

### Participants

A total of 507 native Italian speakers were enrolled to participate in an online study (436 females and 81 males; mean age: 20.82 years, *SD* = 2.22; mean education: 15.16 years, *SD* = 1.11). Participants were either recruited through social networks or identified via researchers’ personal networks. Participants completed an online informed consent form prior to completing the survey. The procedure used in the study is in accordance with the ethical standards of the 2013 Declaration of Helsinki for human studies of the World Medical Association and was approved by the Departmental Ethics Committee of the University of Padua.

### Materials

We selected 1,957 Italian words from our Italian adaptations of the original ANEW (Montefinese et al., 2014; Fairfield et al., 2017) and from available Italian semantic norms (Zannino et al., 2006; Kremer and Baroni, 2011; Montefinese et al., 2013a,b). The selection of words was intended to provide researchers with normative data for a large set of words, for which other lexical-semantic variables are available. The set of stimuli included 76% of nouns, 16% of adjectives, and 8% of verbs. The word stimuli were presented in the same verbal form as the previous Italian norms (e.g., the verbs were presented in the infinitive form) to preserve the consistency with these data collections (Montefinese et al., 2014; Fairfield et al., 2017). There was a higher number of nouns because the other affective-semantic variables (from the other Italian norms) were available especially for nouns compared to the verbs and adjectives.

Word stimuli were distributed over 20 lists containing 97–98 words each. In order to avoid primacy or recency effects, the order in which words appeared in the list was randomized for each participant separately. All lists were roughly matched for word length, word frequency, number of orthographic neighbors, and mean frequency of orthographic neighbors.

### Procedure

For each list, an online form was created using Google modules. Participants who agreed to participate in the study received the link to complete the survey from any electronic device with access to the Internet. Participants were asked to estimate the age (in years) at which they thought they had learned the word, specifying that this information should indicate the age at which they understood the word when somebody else used it in their presence for the first time, even when they did not use the word themselves. These instructions and the examples provided to the participants closely matched those used in a large number of previous studies (Ghyselinck et al., 2000; Stadthagen-Gonzalez and Davis, 2006; Kuperman et al., 2012; Moors et al., 2013; Łuniewska et al., 2016). We included the original Italian instructions and their English translation in the Instructions sheet of the ItAoA.xlsx file.

The task lasted about 40 min.

## Results

### Database

The normative data include values of AoA for 1,957 Italian words provided by 507 native Italian speakers (see section “Participants”). Each word was rated by 25 participants. There were a few missing values because some word meanings were unknown to a few participants (0.3% of the total responses). The database includes the full list of Italian words, their English translations, the corresponding AoA estimates, log-transformed word frequency measures derived from different data sources (“La Repubblica” corpus, Baroni et al., 2004; Baroni and Kilgarriff, 2006; ItWaC corpus, Baroni et al., 2009; Baroni and Kilgarriff, 2006; Subtlex corpus^1^) and neighborhood density^2^ and orthographic similarity (Yarkoni et al., 2008) dimensions. The content of the database (including a description of variables and their related references) is described in more detail in the Description sheet of the ItAoA.xlsx file. The ItAoA norms are freely available to the scientific community for non-commercial use at Open Science Framework repository^3^. Table 1 presents descriptive statistics for all of the variables included in the database.

**Table 1:** Descriptive statistics for the measures included in the ItAoA database.

	Nouns				Adjectives				Verbs				Total			
Measures	*M*	*SD*	Min	Max	*M*	*SD*	Min	Max	*M*	*SD*	Min	Max	*M*	*SD*	Min	Max
M_AoA	6.5	2.2	1.9	14.4	7.4	2.2	3.1	13.3	5.6	2	2.8	12.4	6.6	2.2	1.9	14.4
SD_AoA	2.3	0.7	0.8	5.4	2.5	0.6	0.9	4.2	2.2	0.6	0.8	4.2	2.3	0.7	0.8	5.4
#Letters	6.9	1.9	2	16	7.6	2	3	13	8.1	1.8	4	14	7.1	2	2	16
WF_La Repubblica	7.9	2	0	13.8	8.1	2	0.7	13.3	9.2	2.2	1.6	14.2	8	2.1	0	14.2
WF_ItWaC	6.9	1.7	0	11.9	7	1.9	1.6	12.5	8.5	2.3	1.9	14	7.1	1.9	0	14
WF_Subtlex	6.6	1.9	0	11.8	6.7	2	0	12.6	7	2.2	1.9	12.3	6.6	2	0	12.6
#OrtNB	4.2	5.1	0	34	4.1	3.3	0	19	2.8	3.7	0	26	4.1	4.7	0	34
M_WF_OrtNB	1.8	1.3	0	6.8	1.9	1.2	0	5.7	1.3	1.1	0	4.5	1.8	1.3	0	6.8
MaxWF_OrtNB	2.4	1.9	0	9.3	2.5	1.6	0	8.5	1.7	1.6	0	6.9	2.4	1.8	0	9.3
OLD20_Subtlex	2.1	0.6	1	5.4	2	0.5	1	4.1	1.7	0.2	1	2.8	2	0.6	1	5.4

*M, mean; AoA, age of acquisition; SD, standard deviation; #, number; WF, log-transformed word frequency; OrtNB, orthographic neighbors; OLD20, Orthographic Levenshtein Distance 20. See the Description sheet in the ItAoA.xlsx file.*

### Descriptive Statistics

**FIGURE 1:**
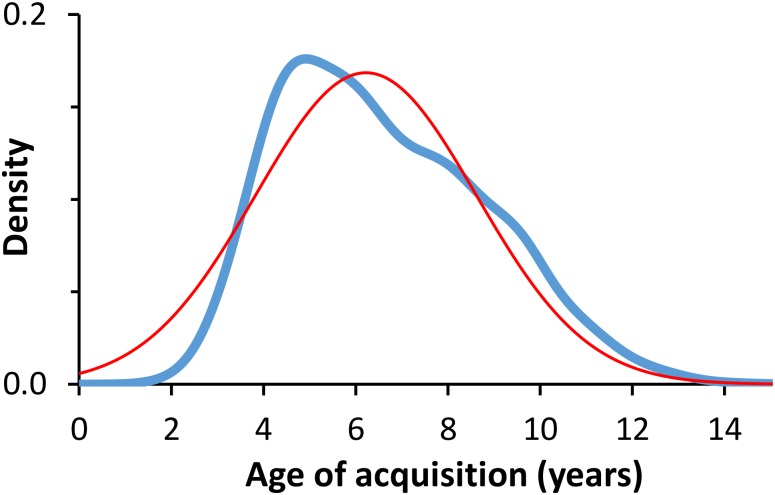
Distribution of mean AoA ratings. The figure shows the probability density estimate of the mean AoA ratings (blue line) based on a normal kernel function. The best-fitting Gaussian (normal) density function is also shown in red for reference.

**FIGURE 2:**
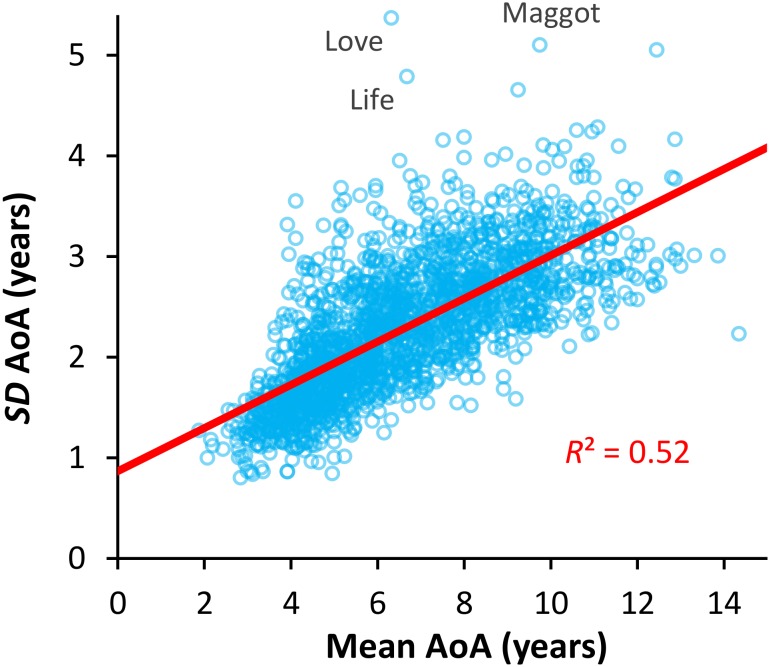
Relation between mean and SD for the AoA ratings. Mean AoA ratings are plotted against the corresponding SDs values. The best-fitting linear regression line is shown in red. The labeled points represent the three words with abnormally high residuals (both raw and deleted residuals > 2; standardized residuals > 4).

Figure 1 shows the distribution of the mean ratings of AoA for all participants. The distribution deviated significantly from a normal distribution (Kolmogorov–Smirnov test: *d* = 0.075, *p* = 0.01) with a mean of 6.61 years (median = 6.28 years), an *SD* of 2.21 years (*IQR* = 3.4 years), and data points ranging between 1.88 and 14.35 years. Kurtosis was -0.43 (*SE* = 0.11), indicating a relatively flat distribution compared to the normal model, and the skewness was slight positive (0.49, *SE* = 0.11). With regards to the homogeneity of the participants’ ratings, Figure 2 shows the means of the ratings for each word plotted against their standard deviations for the AoA variable. From inspecting the scatterplot, it may be noted that the *SD*s increase with the increase of the means. This impression was corroborated by a regression analysis (*r* = 0.72, *F*_(1,1955)_ = 2079.75, *p* < 0.0001), showing that early-learned words were rated with higher agreement (i.e., low *SD*s) compared with the later-learned words, for which a higher variability (i.e., high *SD*s) has been shown. This is not surprising, since a word can obtain an extremely low mean AoA value only if most of its ratings have very low values, yielding a low *SD*.

A reviewer wondered whether AoA for some words might differ between female and male raters thus affecting our results. As we had a substantially unbalanced distribution of male and female raters (which is quite normal in studies enrolling Psychology students), we did not have enough male participants to most effectively carry out such a comparison. Nonetheless, the correlation between male and female AoA ratings was quite high (*r* = 0.77), suggesting that our results were not affected by the imbalance in the number of male and female participants who rated our words. Moreover, we performed independent-sample *t*-tests for each word, contrasting male vs. female AoA ratings, as well as the corresponding equivalence tests (conservatively using a Cohen’s *d* value of 1; see Lakens, 2017 and Montefinese et al., 2018, for a detailed description of this approach). The results from these tests were inconclusive. Indeed, after false discovery rate (FDR) correction, none of the words in our dataset showed reliable sex-related differences or evidence for a significant equivalence between AoA ratings from male and female raters, thus confirming that further research using larger and more balanced samples of participants is needed to address the issue about gender-related differences in AoA.

### Reliability of the Measure

The consistency of the collected data was first evaluated by applying split-half correlations corrected with the Spearman-Brown formula after randomly dividing the participants into two subgroups of equal size. The reliability indexes were calculated on 2,000 different randomizations of the participants. The corrected split-half correlations were very high [median = 0.953, range = (0.947–0.958)], revealing that the resulting ratings were highly reliable and can be used across the entire Italian-speaking population.

We then assessed the reliability of the resulting norms by examining the correlations between the ratings of our ItAoA norms and the corresponding subjective AoA ratings for the same words in the previous Italian norms (Dell’Acqua et al., 2000; Barca et al., 2002; Rinaldi et al., 2004; Della Rosa et al., 2010; Borelli et al., 2018). In doing this, we converted raw ratings from numeric values into either 7-point (Barca et al., 2002; Della Rosa et al., 2010; Borelli et al., 2018) or 9-point (Dell’Acqua et al., 2000) Likert-like scale values. The estimated reliability of the ratings was very high (*r*s > 0.831, *p*s < 0.001, *r*^2^s > 0.690).

To further assess the validity of our approach we correlated ItAoA ratings with an “objective” index of AoA calculated as the percentage of children in the age range of 16–30 months that can produce a given word as estimated by parents (Rinaldi et al., 2004). The correlation was significant (*N* = 519, *r* = -0.531, *p* < 0.001) with a medium effect size of *r*^2^ = 0.282. Note that this correlation was negative because it is assumed that the higher the percentage of children producing a certain word, the earlier that word is acquired. Moreover, ItAoA ratings were significantly correlated (*N* = 189, *r* = 0.702, *p* < 0.001) with an objective measure of AoA computed as the median value (in months) of the youngest age group that reached a 75% accuracy in a naming task (Lotto et al., 2010). On the basis of all these correlations, we may safely conclude that our ratings are as valid as those previously collected for the Italian language.

To further test the generalizability of our AoA ratings, we correlated them with ratings from previous studies in other languages (Stadthagen-Gonzalez and Davis, 2006; Ferrand et al., 2008; Cameirão and Vicente, 2010; Kuperman et al., 2012; Schock et al., 2012; Moors et al., 2013; Łuniewska et al., 2016).

In particular, we were interested in the correlation between our AoA norms for Italian words, and AoA norms for their translation equivalents in English, which included almost all the words of our database (Kuperman et al., 2012), and between our data and that of Łuniewska et al. (2016) which included AoA ratings for 30 different languages (see Table 2). In the first case, there were 1,319 words in common with Kuperman et al. (2012) and a correlation of *r* = 0.697. In the second case, although there were only 196 words in common the correlations were all significant (*r*s > 0.298, *p*s < 0.001, *r*^2^s > 0.089). It is worth noting that the correlation with the ratings obtained by Italian participants was the highest one (*r* = 0.657, *p* < 0.001, *r*^2^ = 0.432), confirming the results derived from the comparisons with the other AoA ratings for Italian language (Dell’Acqua et al., 2000; Barca et al., 2002; Rinaldi et al., 2004; Della Rosa et al., 2010). All correlations are shown in Table 2.

**Table 2:** Correlation coefficients (Pearson’s *r*) between our age-of-acquisition (AoA) and previous data.

Authors	Language	*N*	*r*	*r*^2^
Barca^a^	Italian	626	0.916	0.838
Borelli^a^	Italian	94	0.941	0.885
Della Rosa	Italian	202	0.903	0.815
Dell’Acqua^b^	Italian	252	0.831	0.690
Rinaldi^c^	Italian	519	-0.531	0.282
Lotto^d^	Italian	189	0.702	0.493
Schock^a^	English	332	0.726	0.527
Cameirão and Vicente^b^	Portuguese	368	0.724	0.525
Stadthagen-Gonzalez and Davis	English	326	0.697	0.486
Kuperman	English	1634	0.697	0.485
Moors	Dutch	1319	0.637	0.406
Ferrand	French	497	0.624	0.390
Luniewska^e^	Italian	195	0.657	0.432
	English	196	0.610	0.372
	South African English	196	0.596	0.355
	Serbian	196	0.579	0.335
	Hebrew	196	0.564	0.318
	American English	195	0.562	0.315
	Western Armenian	169	0.556	0.309
	German	196	0.549	0.301
	Dutch	196	0.545	0.298
	Greek	173	0.512	0.262
	Swedish	196	0.511	0.261
	Polish	173	0.507	0.257
	Czech	195	0.505	0.255
	Lebanese Arabic	174	0.495	0.245
	Afrikaans	173	0.494	0.244
	Finnish	196	0.493	0.243
	Catalan	195	0.487	0.237
	Luxembourgish	196	0.487	0.237
	Slovak	196	0.480	0.230
	Danish	196	0.474	0.224
	Russian	196	0.473	0.224
	Turkish	196	0.468	0.219
	Malay	195	0.454	0.206
	Spanish	196	0.453	0.205
	Maltese	196	0.446	0.199
	Lithuanian	196	0.420	0.177
	Icelandic	196	0.412	0.170
	Hungarian	196	0.396	0.157
	Irish	195	0.387	0.150
	IsiXhosa	196	0.298	0.089

*^a^7-point Likert scale; ^b^9-point Likert scale; ^c^percentage of children between 16 and 30 months who can produce each word (as estimated by parents); ^d^median value (in months) of the youngest age group with 75% of correct naming responses; ^e^continuous estimate in the 1–18 years range.*

### Relations Among Variables

The matrices of zero-order and partial correlations among the measures for our Italian sample are reported in Tables 3, 4, respectively. The FDR correction was applied at *p* = 0.05, with the procedure described by Benjamini and Hochberg (1995), to correct for multiple comparisons. To avoid problems of excessive multicollinearity among the independent variables, we used only a single measure of written word frequency (ItWaC; Baroni and Kilgarriff, 2006; Baroni et al., 2009). Zero-order pairwise correlations showed that AoA is related to all the variables included in this study. However, when the effects of other variables are partialled out, partial pairwise correlation analysis showed that only seven of the eleven lexical-semantic measures significantly correlated with the AoA. In particular, AoA showed a medium negative correlation with word frequency (*r* = -0.370), a medium-small negative correlations with familiarity (*r* = -0.201) and imageability (*r* = -0.214), and a very small correlation with the mean frequency of use of the orthographic neighbors (*r* = -0.069). Together, these findings suggest that words with higher frequency, familiarity, imageability and with more frequent orthographic neighbors tend to be learned earlier in life. Moreover, AoA had a small positive correlation with arousal (*r* = 0.181) and very small positive correlations with orthographic Levenshtein distance 20 (*r* = 0.084) and dominance (*r* = 0.072). Thus, words with higher values of arousal, lexical similarity and dominance tend to be learned later in life.

**Table 3:** Zero-order correlations (Pearson’s *r*) between all the variables.

Measures^a^		Correlations^b^											
		1	2	3	4	5	6	7	8	9	10	11	12
1	AoA		**<0.001**	**<0.001**	**<0.001**	**<0.001**	**<0.001**	**<0.001**	**<0.001**	**<0.001**	**<0.001**	**<0.001**	**<0.001**
2	#Lett	**0.204**		**<0.001**	**<0.001**	**<0.001**	**<0.001**	**<0.001**	**<0.001**	**<0.001**	**0.001**	**<0.001**	**0.149**
3	WFr	**-0.427**	**-0.290**		**<0.001**	**<0.001**	**<0.001**	**<0.001**	**<0.001**	**0.044**	**<0.001**	**0.011**	**0.000**
4	#OrtNB	**-0.227**	**-0.635**	**0.267**		**<0.001**	**<0.001**	**<0.001**	**<0.001**	**<0.001**	**<0.001**	**<0.001**	**0.020**
5	WFr_OrtNB	**-0.262**	**-0.466**	**0.478**	**0.523**		**<0.001**	**<0.001**	**<0.001**	**<0.001**	**<0.001**	**0.150**	**0.001**
6	OLD20	**0.221**	**0.708**	**-0.380**	**-0.616**	**-0.471**		**<0.001**	**0.019**	0.374	**0.034**	0.627	0.603
7	Fam	**-0.606**	**-0.181**	**0.338**	**0.204**	**0.242**	**-0.131**		**<0.001**	**<0.001**	**<0.001**	**<0.001**	**<0.001**
8	Ima	**-0.582**	**-0.209**	**0.157**	**0.164**	**0.144**	**-0.070**	**0.650**		**<0.001**	**<0.001**	**<0.001**	**<0.001**
9	Con	**-0.478**	**-0.190**	**0.060**	**0.136**	**0.103**	-0.027	**0.504**	**0.881**		**<0.001**	**<0.001**	**0.024**
10	Val	**-0.298**	**-0.095**	**0.211**	**0.124**	**0.164**	**-0.063**	**0.453**	**0.231**	**0.128**		**<0.001**	**<0.001**
11	Aro	**0.278**	**0.155**	**0.076**	**-0.121**	**-0.043**	0.015	**-0.295**	**-0.289**	**-0.342**	**-0.294**		**<0.001**
12	Dom	**-0.195**	**-0.043**	**0.164**	**0.070**	**0.097**	-0.016	**0.383**	**0.151**	**0.067**	**0.849**	**-0.194**	

*^a^AoA, age of acquisition; #Lett, number of letters; WFr, ItWac word frequency; #OrtNB, number of orthographic neighbors; WFr_OrtNB, mean frequency of orthographic neighbors; OLD20, orthographic Levenshtein distance 20; Fam, familiarity; Ima, imageability; Con, concreteness; Val, valence; Aro, arousal; Dom, dominance; see text for details; ^b^correlations are shown in the lower triangle with corresponding p-values in the upper triangle. Significant correlations (α = 0.05, FDR-corrected) are indicated in bold.*

**Table 4:** Partial correlations (Pearson’s *r*) between all the variables.

Measures^a^		Partial correlations^b^											
		1	2	3	4	5	6	7	8	9	10	11	12
1	AoA		0.094	**<0.001**	0.519	**0.022**	**0.005**	**<0.001**	**<0.001**	0.741	0.049	**<0.001**	**0.017**
2	#Lett	-0.050		**<0.001**	**<0.001**	**0.004**	**<0.001**	**0.002**	0.053	0.070	0.403	**<0.001**	0.572
3	WFr	**-0.370**	**-0.159**		0.087	**<0.001**	0.302	**<0.001**	0.033	0.046	0.306	**<0.001**	0.657
4	#OrtNB	-0.019	**-0.233**	-0.052		**<0.001**	**<0.001**	0.381	0.894	0.666	0.896	0.114	0.868
5	WFr_OrtNB	**-0.069**	**-0.087**	**0.292**	**0.305**		0.107	0.409	0.074	0.238	0.038	0.340	0.101
6	OLD20	**0.084**	**0.500**	-0.031	**-0.301**	-0.049		0.466	0.439	**0.019**	0.692	**0.001**	0.228
7	Fam	**-0.201**	**0.094**	**0.140**	0.026	0.025	-0.022		**<0.001**	**<0.001**	**<0.001**	**<0.001**	**<0.001**
8	Ima	**-0.214**	-0.058	-0.064	-0.004	-0.054	0.023	**0.379**		**<0.001**	**0.006**	**<0.001**	0.045
9	Con	0.010	-0.055	-0.060	0.013	0.036	**0.070**	-0**.127**	**0.817**		**<0.001**	**<0.001**	0.350
10	Val	-0.059	0.025	0.031	0.004	0.062	-0.012	**0.086**	**0.082**	-0**.106**		**<0.001**	**<0.001**
11	Aro	**0.181**	**0.160**	**0.257**	-0.048	0.029	**-0.099**	**-0.114**	**0.168**	**-0.220**	**-0.217**		**0.001**
12	Dom	**0.072**	-0.017	0.013	0.005	-0.049	0.036	**0.111**	-0.060	0.028	**0.816**	**0.098**	

*^a^AoA, age of acquisition; #Lett, number of letters; WFr, ItWac word frequency; #OrtNB, number of orthographic neighbors; WFr_OrtNB, mean frequency of orthographic neighbors; OLD20, orthographic Levenshtein distance 20; Fam, familiarity; Ima, imageability; Con, concreteness; Val, valence; Aro, arousal; Dom, dominance; see text for details; ^b^partial correlations are shown in the lower triangle with corresponding p-values in the upper triangle. Significant correlations (α = 0.05, FDR-corrected) are indicated in bold.*

## Discussion

In the present article, we have described a dataset including AoA ratings for 1,957 Italian content words (adjectives, nouns, and verbs), obtained by asking adult participants to estimate the age at which they thought they had learned the word. This study goes beyond previous studies for the Italian language because we obtained AoA ratings for a larger set of words, covering more grammatical categories, and specifically ensuring that AoA ratings were obtained for words for which normative data on other lexical variables are available (Zannino et al., 2006; Kremer and Baroni, 2011; Montefinese et al., 2013b, 2014; Fairfield et al., 2017). A large number of studies showed that AoA is one of the most important variables in predicting performance in healthy participants and patients (Brysbaert et al., 2000b; Ellis and Lambon Ralph, 2000; Barry and Gerhand, 2003; Weekes et al., 2003; Sartori et al., 2005; Lambon Ralph and Ehsan, 2006; Cortese and Khanna, 2007; Navarrete et al., 2013). For this reason, the availability of AoA ratings for a large number of words is particularly important because it will allow researchers to manipulate and control this variable in future research.

An exploration of the distribution of AoA ratings revealed that their distribution deviated significantly from the normality and the ratings’ variability increased with the increase of the mean of AoA, suggesting that participants learn similar words early in their life and show more variability in later years. Although this result can be different in size, it is quite consistently significant across several languages: Portuguese (Marques et al., 2007), Dutch (Moors et al., 2013), Icelandic (Pind et al., 2000), Italian (Barca et al., 2002), and French (Alario and Ferrand, 1999). In contrast, Cameirão and Vicente (2010) found a negative correlation between the mean of AoA ratings and their standard deviations. This discrepancy in the results could be due to several factors. By plotting the mean and standard deviations of Cameirão and Vicente’s ratings, it is possible to infer that the pattern of results is better described by a quadratic function, suggesting that their participants agreed more in the AoA estimates of both later- and earlier-acquired words. This is likely due to the fact that Cameirão and Vicente used a Likert scale, so words that obtained either extremely low or extremely high mean AoA value were mathematically forced to have a low *SD*. Moreover, it is worth noting that, unlike our ItAoA norms, a high percentage of the word stimuli (70% of the total set) in Cameirão and Vicente’s set were rated as having high AoA, thus driving the negative correlation they found between the mean of AoA ratings and their standard deviations.

We evaluated the reliability of our ItAoA norms in three different manners. First, we established their internal consistency as shown by the high split-half correlations between 2,000 random subsets of participants of our sample, suggesting a large agreement among our participants. Second, their validity was confirmed in comparisons with subjective AoA ratings for Italian in adults (Dell’Acqua et al., 2000; Barca et al., 2002; Della Rosa et al., 2010; Borelli et al., 2018) and objective AoA measures in children (Rinaldi et al., 2004). As in previous studies (Morrison et al., 1997; Ghyselinck et al., 2000; Pind et al., 2000), this last comparison allowed us to confirm the validity of using adult estimations for AoA. Finally, as for AoA norms in other languages (Stadthagen-Gonzalez and Davis, 2006; Ferrand et al., 2008; Cameirão and Vicente, 2010; Kuperman et al., 2012; Schock et al., 2012; Moors et al., 2013; Łuniewska et al., 2016), our results revealed significant cross-linguistic correlations, suggesting strong cross-language stability of our data.

We also investigated the pattern of relations among AoA and other lexical-semantic variables. Overall, correlations of AoA with the other dimensions are similar to those obtained in previous studies in different languages (Bird et al., 2001; Barca et al., 2002; Stadthagen-Gonzalez and Davis, 2006; Marques et al., 2007). In particular, in all these studies, word frequency, familiarity and imageability are the variables more consistently related to AoA. Thus, earlier-acquired words tend to be more frequent, familiar and imageable than later-acquired words. However, regarding affective variables our correlational results are only partially congruent with those of Moors et al. (2013) who found a negative correlation between AoA and valence and a positive correlation between AoA and dominance, suggesting that early-acquired words are positive and more dominant. Our zero-order correlations confirmed the negative relation between AoA and valence (but which did not survive correction for multiple comparisons in the partial correlation analysis), but unlike Moors et al. (2013) norms, they also revealed a positive relation between AoA and arousal, as well as a negative relation between AoA and dominance (which, however, reversed in the partial correlation analysis). A possible factor contributing to this divergence could be the different type of instructions adopted by Moors et al. (2013), who asked participants to rate the active/dominant meaning of the stimuli, whereas we asked participants to rate their own feelings of arousal/dominance in response to the stimuli.

Moreover, as in Marques et al. (2007) and Cameirão and Vicente (2010), the number of letters was not a significant predictor of AoA ratings and the pattern of correlation between AoA and word orthographic similarity (operationalized as OLD20; Yarkoni et al., 2008) we reported is compatible with previous findings (Kuperman et al., 2012).

In sum, in the present study, we collected the AoA norms for 1,957 Italian content words from three distinct grammatical classes: adjectives, nouns, verbs. We showed significant correlations between the AoA and other lexical-semantic variables, such as word frequency, imageability, familiarity and dominance in line with the literature. Moreover, the high reliability and validity has been demonstrated by high correlations between ItAoA ratings and the other Italian AoA norms collected in children and adults. We also showed their high across-language consistency by comparing ItAoA ratings with the ones available for English, French, Dutch, and so forth. We believe that ItAoA norms are a valuable source of information and can be used confidently for the selection of words in future research.

## Data Availability Statement

The datasets for this study can be found at the Open Science Framework repository (https://osf.io/3trg2/).

## Author Contributions

MM and EA contributed to the conception and design of the study, performed the statistical analysis, and organized the database. MM wrote the first draft of the manuscript. All authors contributed to manuscript revision, read and approved the submitted version.

## Conflict of Interest Statement

The authors declare that the research was conducted in the absence of any commercial or financial relationships that could be construed as a potential conflict of interest.

## Footnotes

1 http://crr.ugent.be/subtlex-it

2 http://ip146172.psy.unipd.it/claudio/vicini2.php

3 osf.io/3trg2
